# Supplementary figures and images for: Imprecise recombinant viruses evolve via a fitness-driven, iterative process of polymerase template-switching events
Source: PLoS Pathog. 2021 Aug 20;17(8):e1009676. doi: 10.1371/journal.ppat.1009676 (PMC8409635; doi:10.1371/journal.ppat.1009676)

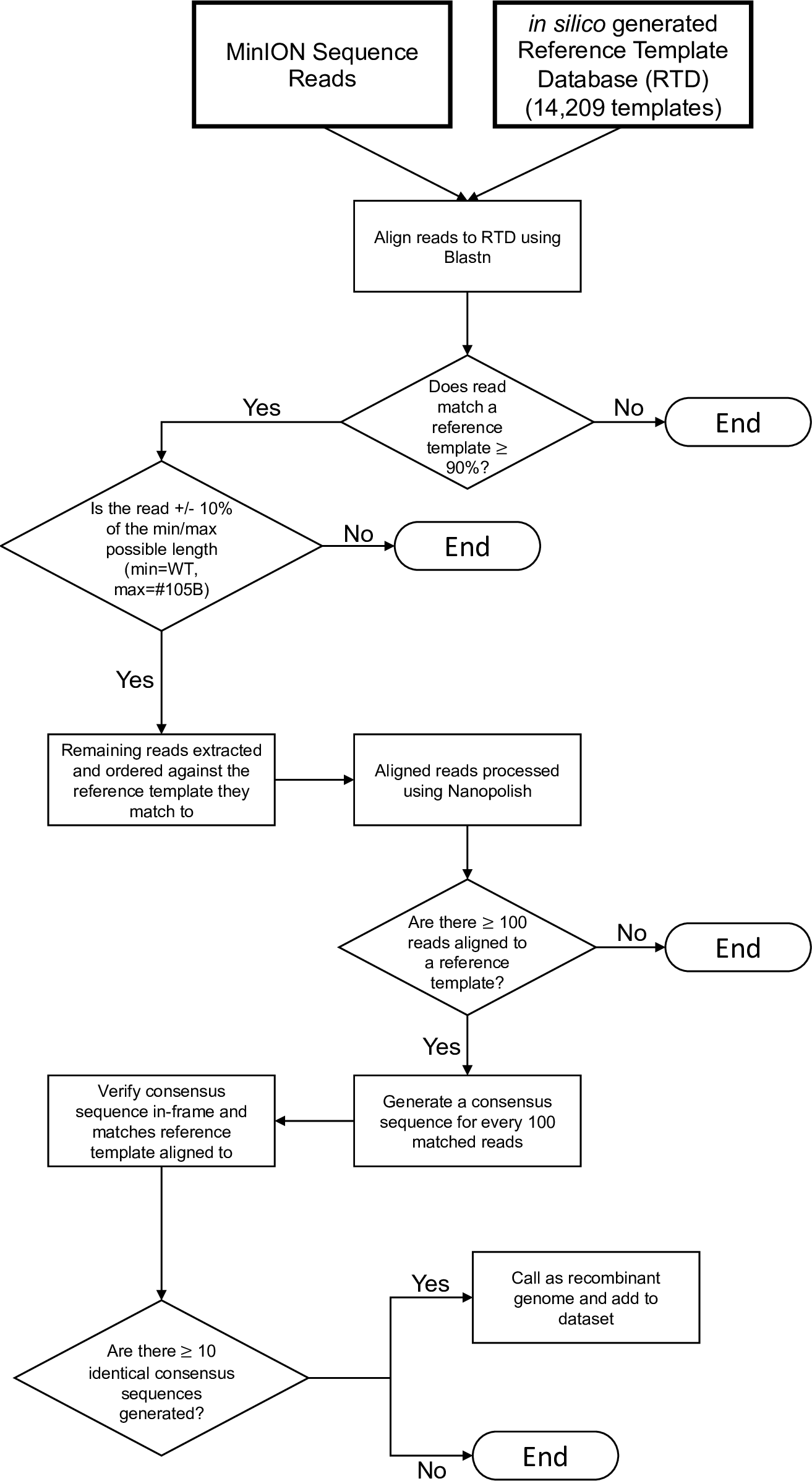

Supplement: S1 Fig — Flowchart shows each step taken to generate recombinant sequences from Oxford Nanopore sequencing data. Boxes represent processes in the pipeline, diamonds represent decision points and ovals represent termination points. (TIF) [file ppat.1009676.s001.tif]

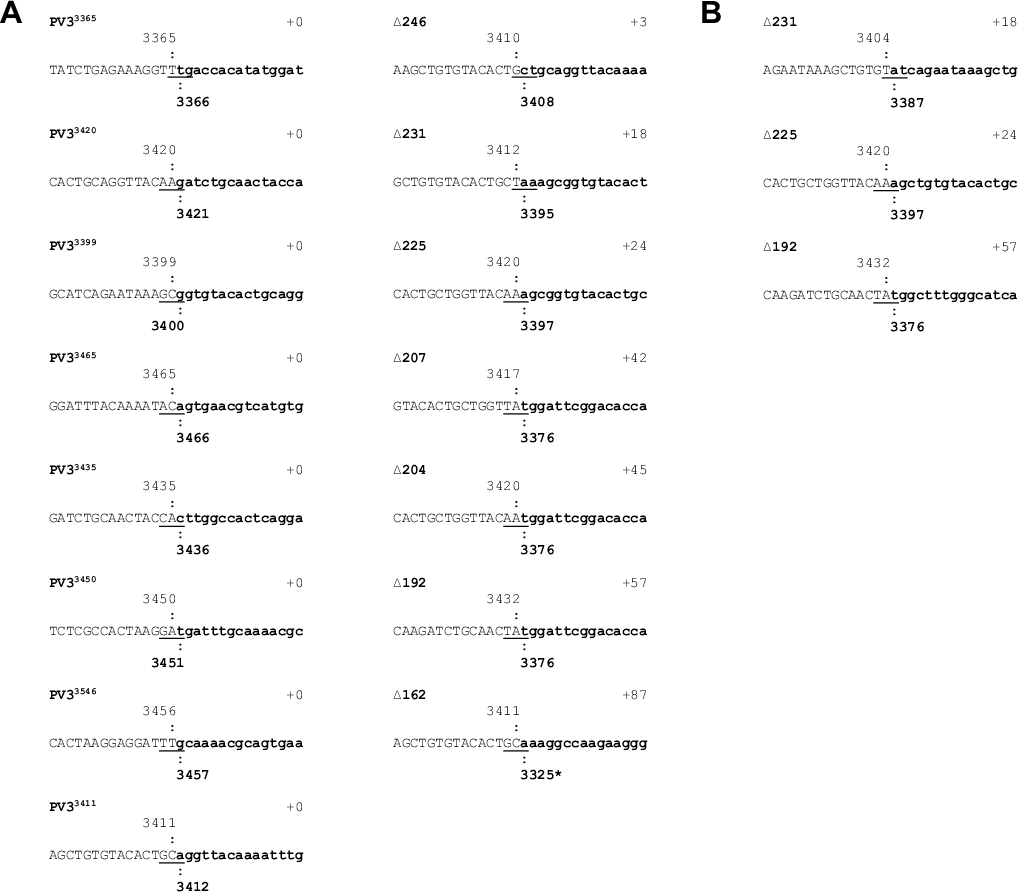

Supplement: S2 Fig — Sequences denote the 30 nt spanning the junction of individual #105B (A) or #PV3-105B (B) resolving recombinants (designation in upper left corner). Uppercase sequences distinguish PV3 derived sequence from PV1 replicon derived sequence with numbers denoting the genome positions relative to full length PV3 and PV1. Numbers in the upper right corner indicate the number of additional nucleotides present in the recombinant genome, while underlined nucleotides indicate the reading-frame of the recombinant. (TIF) [file ppat.1009676.s002.tif]

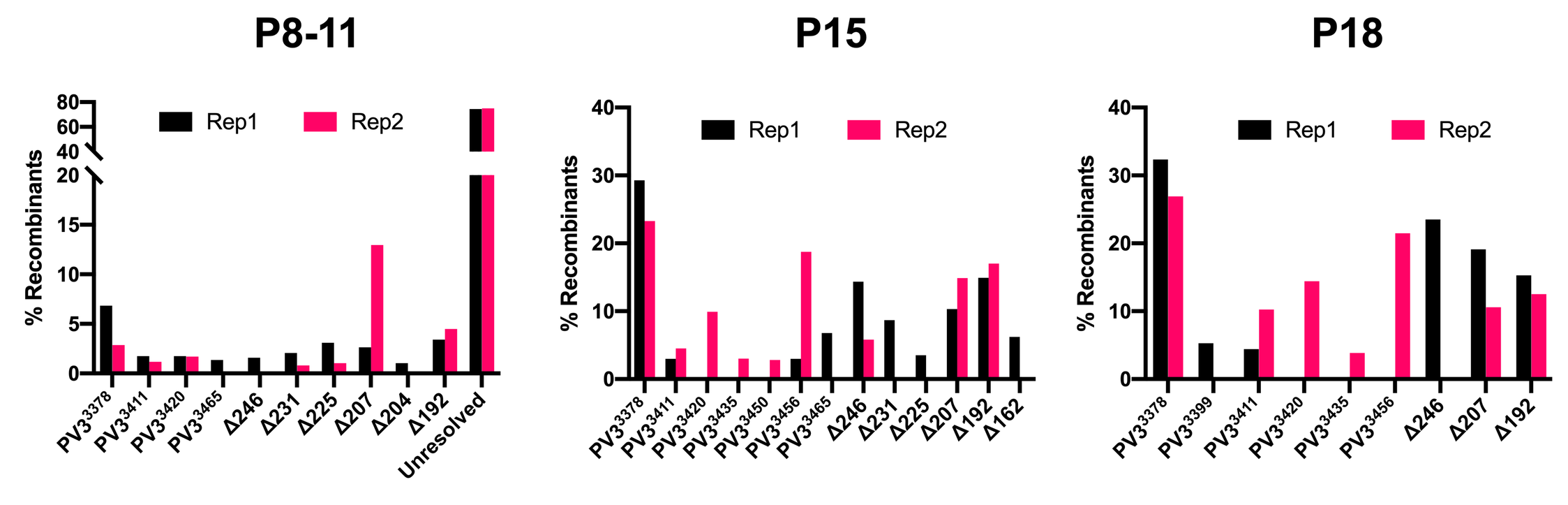

Supplement: S3 Fig — The frequency of each variant within the overall population was determined based on the number of consensus sequences generated for each variant. The number of consensus sequences for each variant was expressed as a percentage of the total number of consensus sequences generated at each passage (or pooled passages). Variants are shown for both Replicate 1 (black) and Replicate 2 (pink) passaging. (TIF) [file ppat.1009676.s003.tif]

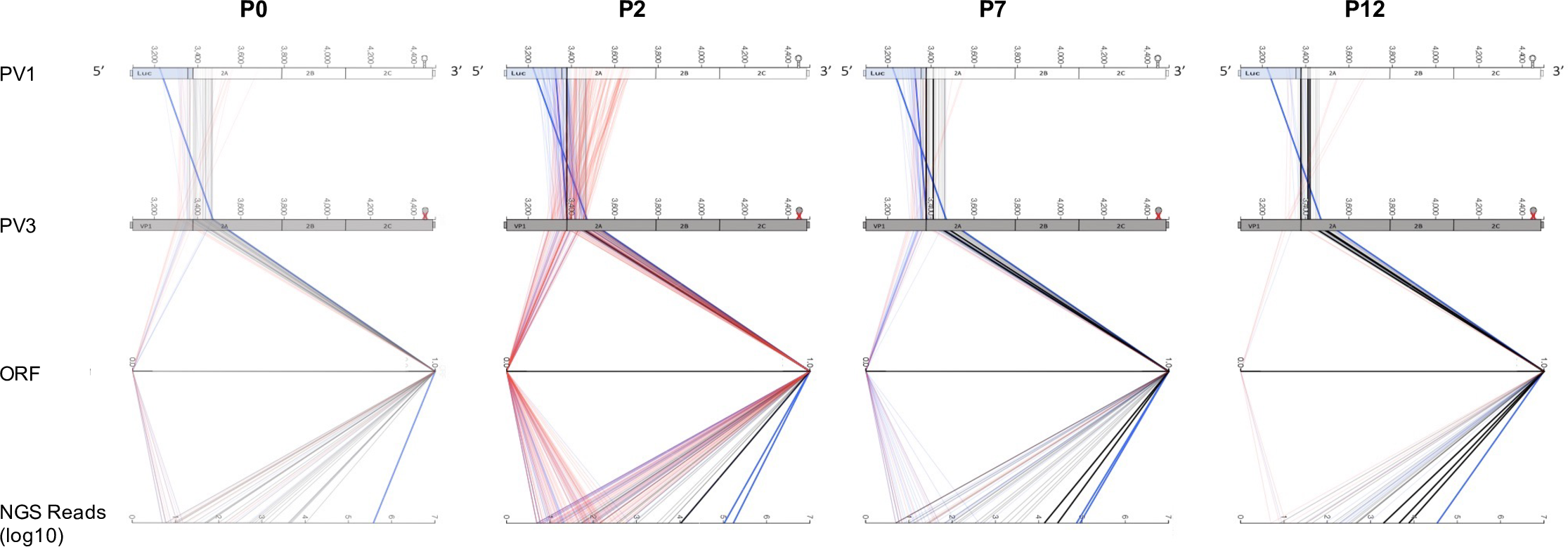

Supplement: S4 Fig — The location of each recombination junction was mapped respective to each parental genome. Each line represents a unique recombinant within the population of precise (black), imprecise-insertion (blue) and imprecise-deletion (red) recombinants. Each recombinant is shown as in-frame (1.0) or out-of-frame (0.0) with log10 read count below. (TIF) [file ppat.1009676.s004.tif]

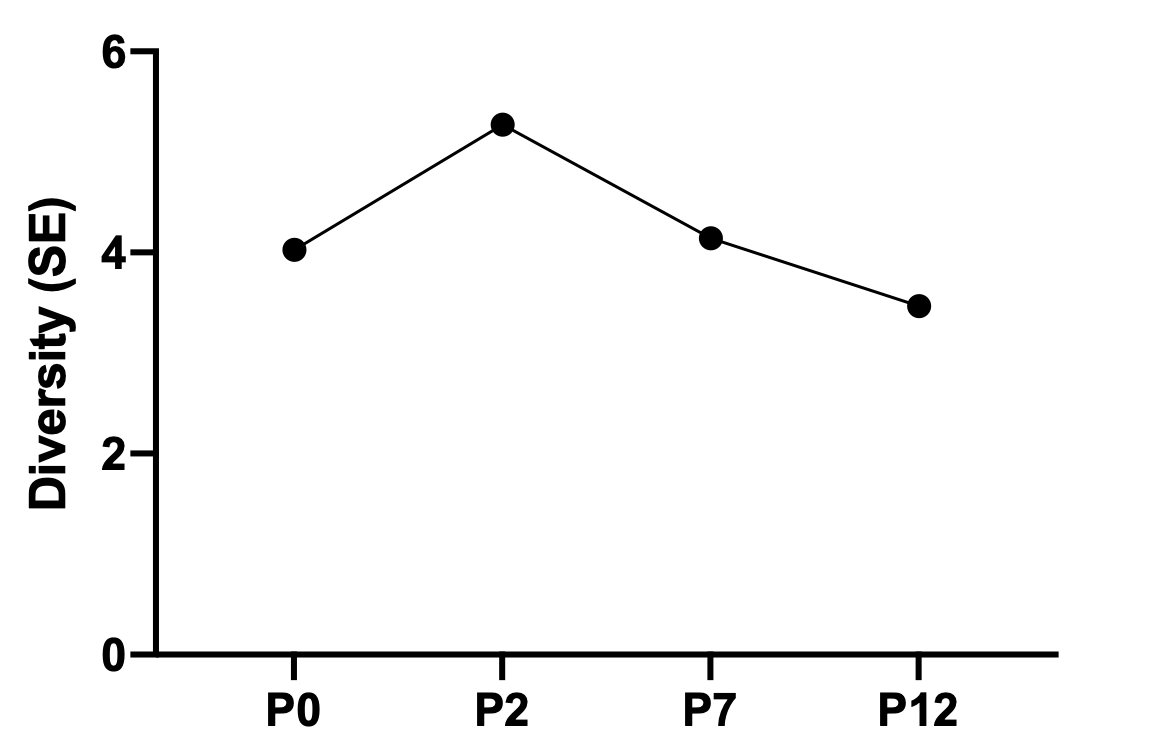

Supplement: S5 Fig — The flattened data was used to calculate Shannon Entropy using the equation H(X)=∑i=0N−1Pilnpi. The probability of each unique junction was calculated by dividing 1 over the total number of junctions, and plotted against passage number. (TIF) [file ppat.1009676.s005.tif]

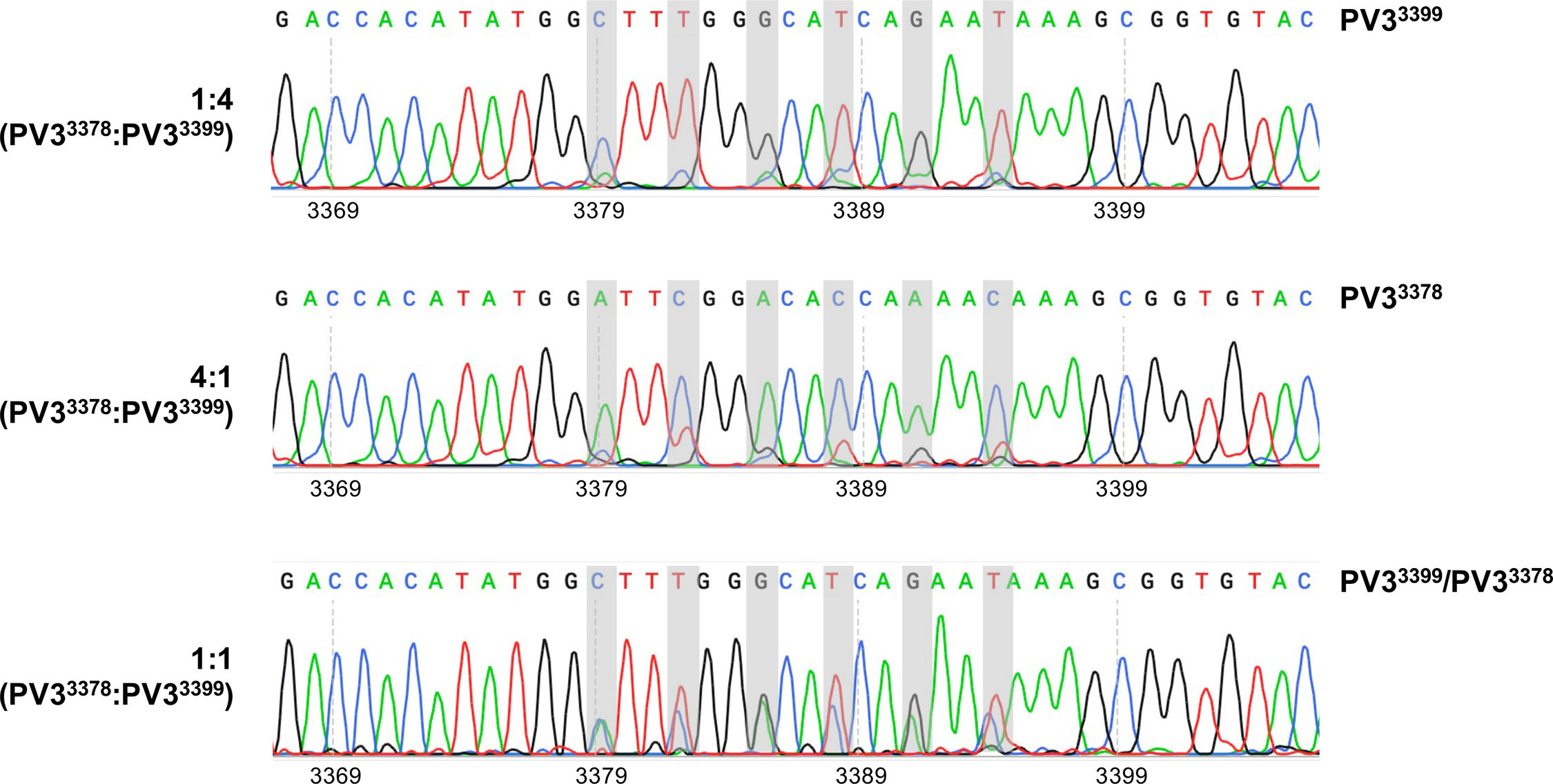

Supplement: S6 Fig — Sequence trace analysis of the viral population following co-infection and 10 passages of 2 resolved variants, PV33378 and PV33399. Viruses were co-infected at ratios of 1:4 (PV33378: PV33399) (upper panel), 4:1 (PV33378: PV33399) (middle panel) and 1:1 (bottom panel) with traces showing the population sequence at p10. Grey boxes highlight the different nucleotides between PV33378 and PV33399 used to determine the dominant sequence in the population. (TIF) [file ppat.1009676.s006.tif]
