# Supplementary material for: Imprecise recombinant viruses evolve via a fitness-driven, iterative process of polymerase template-switching events
Source: PLoS Pathog. 2021 Aug 20;17(8):e1009676. doi: 10.1371/journal.ppat.1009676 (PMC8409635; doi:10.1371/journal.ppat.1009676)
Supplement: S1 Table — (DOCX) [file ppat.1009676.s007.docx]

| **Population** | **Recombinant** | **Passage** | **Ratio** | **Total Reads** |
| --- | --- | --- | --- | --- |
| Unresolved | #105B | p1 | 5% | 32,189 |
|  | #PV3-105B | p1 | 5% | 12,501 |
| Intermediate | #105B | p8-11 | 30% | 115,538 |
|  | #PV3-105B | p10-13 | 30% | 141,487 |
| Resolved | #105B | p15 | 12.5% | 79,560 |
|  | #PV3-105B | p15 | 5% | 31,249 |
|  | #105B | p18 | 12.5% | 62,843 |
