# Supplementary material for: Imprecise recombinant viruses evolve via a fitness-driven, iterative process of polymerase template-switching events
Source: PLoS Pathog. 2021 Aug 20;17(8):e1009676. doi: 10.1371/journal.ppat.1009676 (PMC8409635; doi:10.1371/journal.ppat.1009676)
Supplement: S2 Table — (DOCX) [file ppat.1009676.s008.docx]

| **Variant** | **Duplication (nts)** | **5′ nt** | **3′ nt** | **Ambiguity (nts)** | **Replicates** |
| --- | --- | --- | --- | --- | --- |
| **#105B** | 249 | PV3^3471^ | PV1^3222^ | 0 | 1, 2 |
| **#105B** ∆162 | 87 | PV3^3411^ | PV1^3324^ | 0 | 1 |
| **#105B** ∆192 | 57 | PV3^3432^ | PV1^3375^ | 2 | 1, 2 |
| **#105B** ∆204 | 45 | PV3^3420^ | PV1^3375^ | 1 | 1 |
| **#105B** ∆207 | 42 | PV3^3417^ | PV1^3375^ | 2 | 1, 2 |
| **#105B** ∆225 | 24 | PV3^3420^ | PV1^3396^ | 4 | 1, 2 |
| **#105B** ∆231 | 18 | PV3^3412^ | PV1^3394^ | 0 | 1, 2 |
| **#105B** ∆246 | 3 | PV3^3410^ | PV1^3407^ | 3 | 1, 2 |
| **PV3^3378^** | 0 | PV3^3378^ | PV1^3379^ | 13 | 1, 2 |
| **PV3^3399^** | 0 | PV3^3399^ | PV1^3400^ | 5 | 1 |
| **PV3^3411^** | 0 | PV3^3411^ | PV1^3412^ | 11 | 1, 2 |
| **PV3^3420^** | 0 | PV3^3420^ | PV1^3421^ | 8 | 1, 2 |
| **PV3^3435^** | 0 | PV3^3435^ | PV1^3436^ | 11 | 2 |
| **PV3^3450^** | 0 | PV3^3450^ | PV1^3451^ | 4 | 2 |
| **PV3^3456^** | 0 | PV3^3456^ | PV1^3457^ | 5 | 1, 2 |
| **PV3^3465^** | 0 | PV3^3465^ | PV1^3466^ | 1 | 1 |
| **#PV3-105B** | 249 | PV3^3471^ | PV3^3222^ | 0 | 1 |
| **#PV3-105B** ∆**192** | 57 | PV3^3432^ | PV3^3375^ | 2 | 1 |
| **#PV3-105B** ∆**225** | 24 | PV3^3420^ | PV3^3396^ | 1 | 1 |
| **#PV3-105B** ∆**231** | 18 | PV3^3404^ | PV3^3386^ | 1 | 1 |
