# Supplementary material for: Imprecise recombinant viruses evolve via a fitness-driven, iterative process of polymerase template-switching events
Source: PLoS Pathog. 2021 Aug 20;17(8):e1009676. doi: 10.1371/journal.ppat.1009676 (PMC8409635; doi:10.1371/journal.ppat.1009676)
Supplement: S3 Table — (DOCX) [file ppat.1009676.s009.docx]

| **Variant** | **Duplication (nts)** | **5′ Tag** | **5′ nt** | **3′ nt** | **3′ Tag** | **Ambiguity (nts)** |
| --- | --- | --- | --- | --- | --- | --- |
| **#105B/T2^3420^** | 0 | 105B | PV3^3420^ | PV1^3421^ | T2 | 8 |
| **#T2/105B^3423^** | 0 | T2 | PV3^3423^ | PV1^3424^ | 105B | 2 |
| **#T2/105B**∆**219** | 30 | T2 | PV3^3405^ | PV1^3375^ | 105B | 2 |
| **#105B/T1**∆2**10** | 39 | 105B | PV3^3415^ | PV1^3376^ | T1 | 1 |
| **#T1/105B**∆2**10** | 39 | T1 | PV3^3415^ | PV1^3376^ | 105B | 1 |
| **#105B/T2**∆207 | 42 | 105B | PV3^3417^ | PV1^3375^ | T2 | 2 |
| **#T1/105B**∆207 | 42 | T1 | PV3^3417^ | PV1^3375^ | 105B | 2 |
| **#105B/T2**∆**189** | 60 | 105B | PV3^3396^ | PV1^3336^ | T2 | 2 |
| **#T1/105B**∆**117** | 132 | T1 | PV3^3416^ | PV1^3284^ | 105B | 0 |
